# Simultaneous Elimination of Left Atrial Posterior Wall Atrial Fibrillation and Creation of Mitral Isthmus Block by Ethanol Infusion in the Vein of Marshall

**DOI:** 10.1002/joa3.70177

**Published:** 2025-08-22

**Authors:** Yasuteru Yamauchi, Yuichiro Sagawa, Kaoru Okishige, Tetsuo Sasano, Kazutaka Aonuma

**Affiliations:** ^1^ Department of Cardiology Japan Red Cross, Yokohama City Bay Hospital Yokohama Kanagawa Japan; ^2^ Department of Cardiovascular Medicine Institute of Science Tokyo Tokyo Japan; ^3^ Department of Cardiology Mito Saiseikai General Hospital Ibaraki Japan

**Keywords:** atrial fibrillation, ethanol injection, left atrial posterior wall, mitral isthmus block, non‐pulmonary vein, vein of Marshall

## Abstract

Atrial fibrillation (AF) originating from the left atrial posterior wall recurred repeatedly, resulting in immediate recurrence of AF (IRAF). Ethanol infusion into the vein of Marshall (VOM) successfully terminated the AF and simultaneously achieved complete mitral isthmus block. This dual effect underscores the potential utility of VOM ethanol infusion not only for mitral isthmus ablation but also for targeting non–pulmonary vein (non‐PV) triggers in complex AF cases.

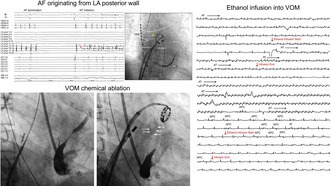

Approximately 20% of non‐pulmonary vein (non‐PV) atrial fibrillation (AF) originates from the left atrial posterior wall (LAPW) [1]. Although radiofrequency (RF) ablation can isolate the LAPW, its success rate is approximately 80%; complete isolation is sometimes difficult. Ethanol infusion into the vein of Marshall (VOM) achieves mitral isthmus block in over 95% of cases. The VOM not only perfuses the mitral isthmus and the left atrial appendage ridge, but may also send small branches to the LAPW [2]. We report a case of successful elimination of LAPW‐origin non‐PV AF and mitral isthmus block via VOM chemical ablation.

A 55‐year‐old man with persistent AF underwent catheter ablation. His echocardiogram showed preserved ejection fraction and a left atrial diameter of 42 mm. In the first procedure, cryoballoon pulmonary vein isolation (PVI) was completed. However, RF‐guided mitral isthmus ablation, including intracoronary sinus ablation, failed to create a mitral isthmus block, and persistent AF recurred immediately post ablation. In the second session, voltage mapping confirmed durable PVI. Despite multiple attempts at electrical cardioversion that temporarily restored sinus rhythm, AF recurred within seconds, indicating immediate recurrence. A duodecapolar ring catheter was placed on the LAPW. Following AF termination, spontaneous AF recurrence was observed, and mapping at AF onset revealed that LAPW potentials recorded by the ring catheter always preceded those in the coronary sinus (CS) and right atrium. The LAPW potentials appeared considerably earlier than the CS and right atrium signals, suggesting that the LAPW was the earliest site of activation. Despite multiple attempts at electrical cardioversion, AF recurred immediately each time, with reproducibly earlier activation in the LAPW region. Furthermore, the local LAPW signals demonstrated continuous, fractionated electrograms, indicating this region as the earliest site of activation (Figure 1). Moreover, during AF, LAPW electrograms exhibited characteristics consistent with complex fractionated atrial electrograms (CFAEs) and a significantly shorter activation cycle than other atrial regions. CFAEs are often linked to non‐PV triggers in persistent AF, and ablation targeting them may be effective beyond PVI. Lo et al. [3] reported that the origin of non‐PV triggers often coincided with the sites of CFAEs and that ablation targeting these CFAEs frequently terminated AF and eliminated the non‐PV triggers. These findings support the presence of AF originating from the LAPW. Therefore, linear ablation on the left atrial roof and floor was performed using a contact‐force sensing irrigated RF catheter (Abbott, St. Paul, MN) to isolate the LAPW; however, complete isolation was not achieved. Despite electrical cardioversion following linear ablation of the LAPW, AF from LAPW immediately recurred, preventing sinus rhythm maintenance. Identifying the conduction gaps along the linear ablation lines was difficult due to ongoing fibrillatory activity within or outside the LAPW. As LAPW isolation was abandoned and mitral isthmus ablation had failed in the first session, we proceeded with chemical ablation of the VOM. Occlusive CS angiography revealed a well‐developed, branching VOM and severe CS stenosis due to previous ablation (Figure 2A). A small VOM branch extended toward the LAPW. A 1.5‐mm balloon catheter with double markers was inserted into the VOM (Figure 2B). After ballooning, 2 mL of 99.5% ethanol was infused over 90 s. Then, 1 mL of contrast agent was injected to check the flow in the VOM (Figure 2C). The balloon was withdrawn slightly, and a second 2‐mL ethanol injection was administered after confirming no leakage (Figure 2D).

**FIGURE 1:**
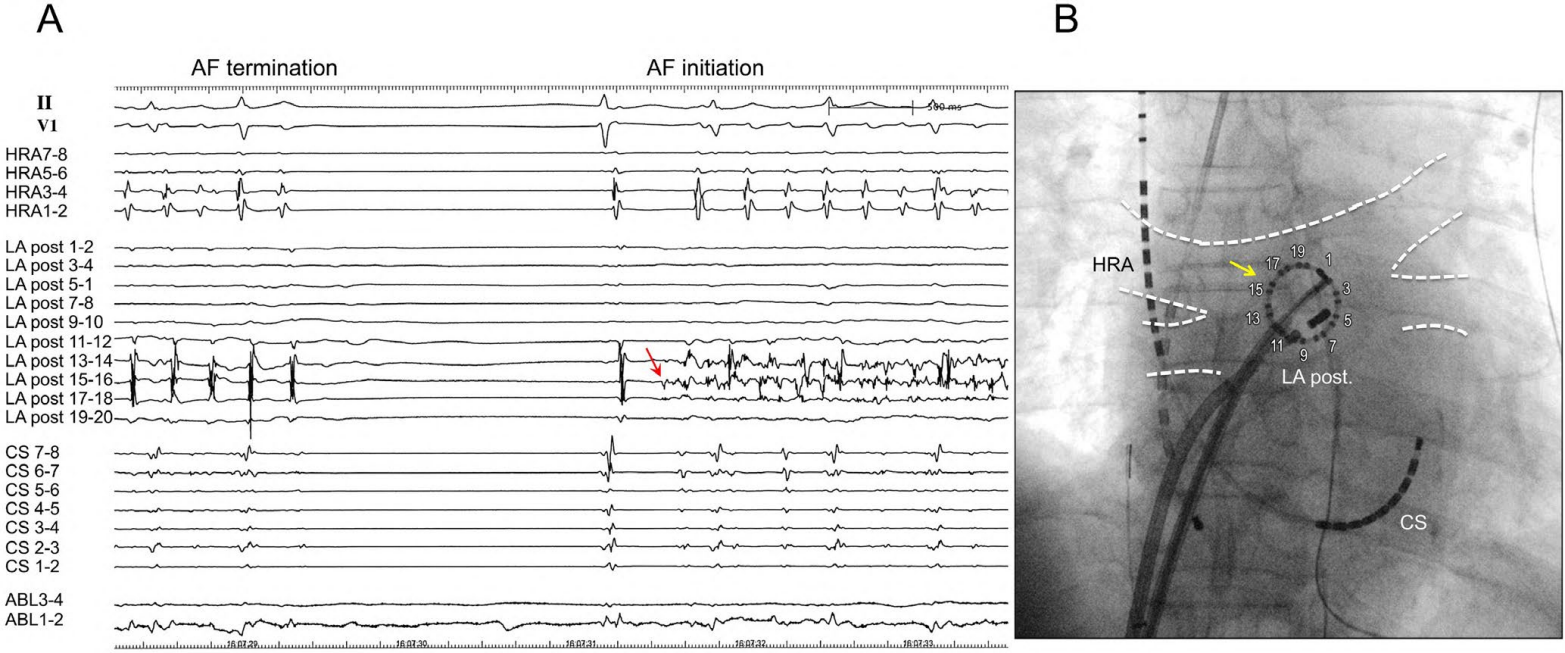
Intracardiac electrograms obtained during non‐PV AF initiation. (A) At the onset of AF, excitation from the ring electrode catheter placed on the left atrial posterior wall (LAPW) was the earliest. Immediately after the onset of AF, the excitation cycle length of the LAPW, which exhibited fractionated potentials, was significantly shorter than that of the CS or HRA potentials. (B) The duodecapolar ring catheter was placed at the center of the LAPW. Dotted lines indicate the positions of each pulmonary vein and the left atrial roof. ABL, ablation catheter; AF, atrial fibrillation; CS, coronary sinus; HRA, high right atrium; LA post, left atrial posterior wall; PV, pulmonary vein.

**FIGURE 2:**
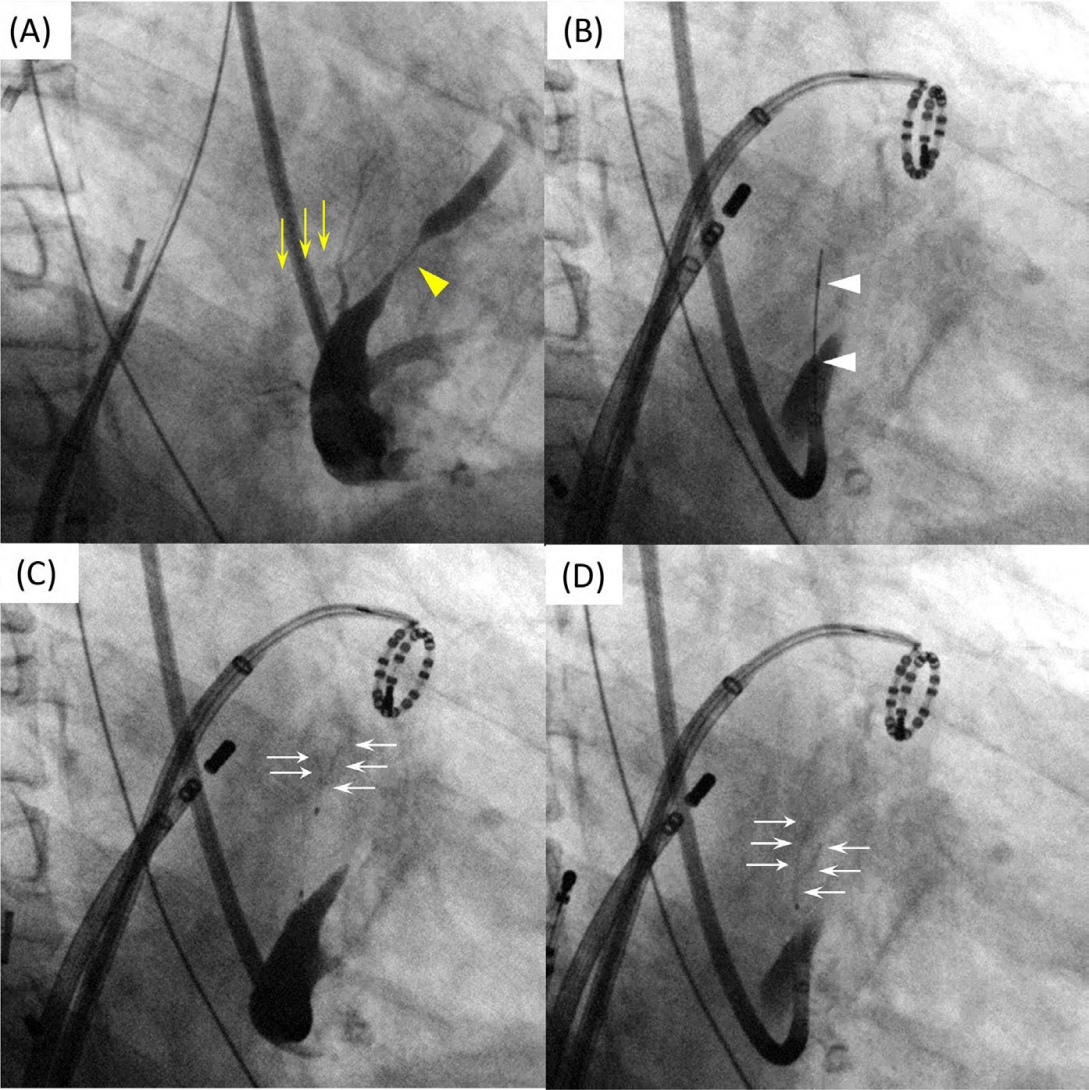
Balloon‐occluded coronary sinus venography and chemical ablation of VOM. (A) Coronary sinus (CS) stenosis is indicated by a yellow arrowhead, and a small branch of the VOM extending toward the LAPW is indicated by yellow arrows. (B) A 1.5 mm diameter balloon with double markers is inserted into the VOM over a guidewire with double markers indicated by white arrowheads. (C) Balloon‐occluded venography showing the VOM and its branches, as indicated by white arrows. (D) After the initial ethanol infusion, the balloon was withdrawn proximally by 2 cm, and balloon‐occluded venography was repeated, highlighting the main and proximal branches of the VOM, as indicated by white arrows. VOM, vein of Marshall; LAPW, left atrial posterior wall.

During the initial ethanol infusion, AF briefly terminated but recurred along with atrial tachycardia (AT) 20 s later (Figure 3). Upon completion of the infusion, both AF and AT ceased. AT then recurred briefly and spontaneously terminated. Thereafter, AF and AT gradually subsided, with only atrial premature contractions (APCs) and short AF episodes observed. Following the second ethanol infusion, the APCs disappeared entirely, allowing stable sinus rhythm. After VOM chemical ablation, a complete mitral isthmus block was achieved (Figure 4). With the restoration and maintenance of sinus rhythm, RF ablation at the gap sites along the left atrial roof and floor lines was insufficient to achieve LAPW isolation; therefore, a centerline ablation was added across the posterior wall, which resulted in successful LAPW isolation (Figure 5). The patient has remained free of atrial arrhythmia recurrence over 5‐year follow‐up.

**FIGURE 3:**
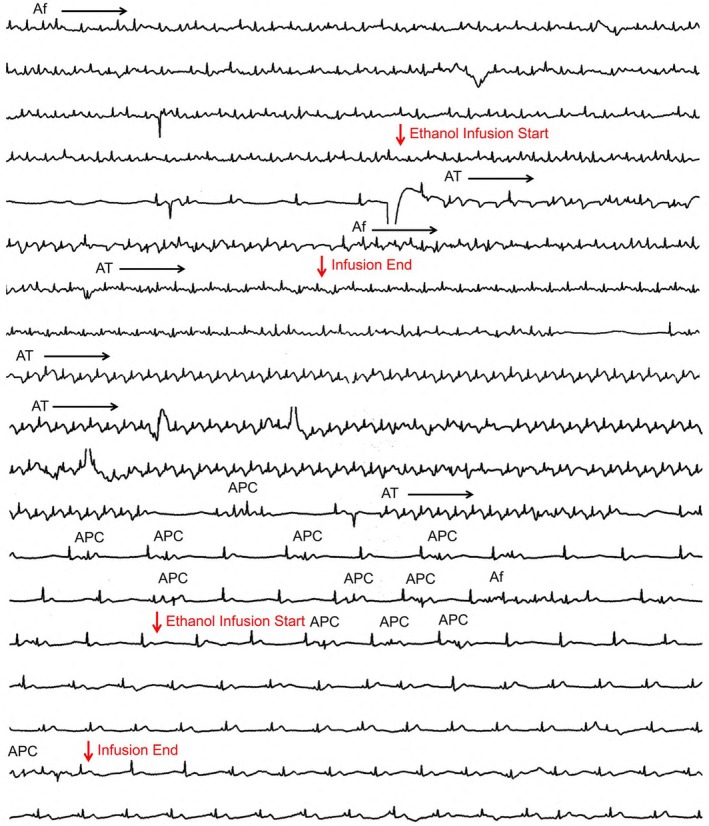
Continuous recording of lead II electrocardiograms during two rounds of VOM chemical ablation. AF was terminated immediately after the start of the initial ethanol infusion, followed by prolonged sinus arrest. Subsequently, AT recurred, degenerated into AF, and subsequently into another AT. The AT degenerated into the AF and then terminated. Immediately after conversion to sinus rhythm, AT recurred but spontaneously terminated. Thereafter, AT and AF did not recur; however, frequent APCs were observed. A second ethanol infusion was initiated during the frequent APCs. After the second ethanol infusion, the APCs completely disappeared, and sinus rhythm was successfully maintained. AF, atrial fibrillation; AT, atrial tachycardia; APC, atrial premature contractions; VOM, vein of Marshall.

**FIGURE 4:**
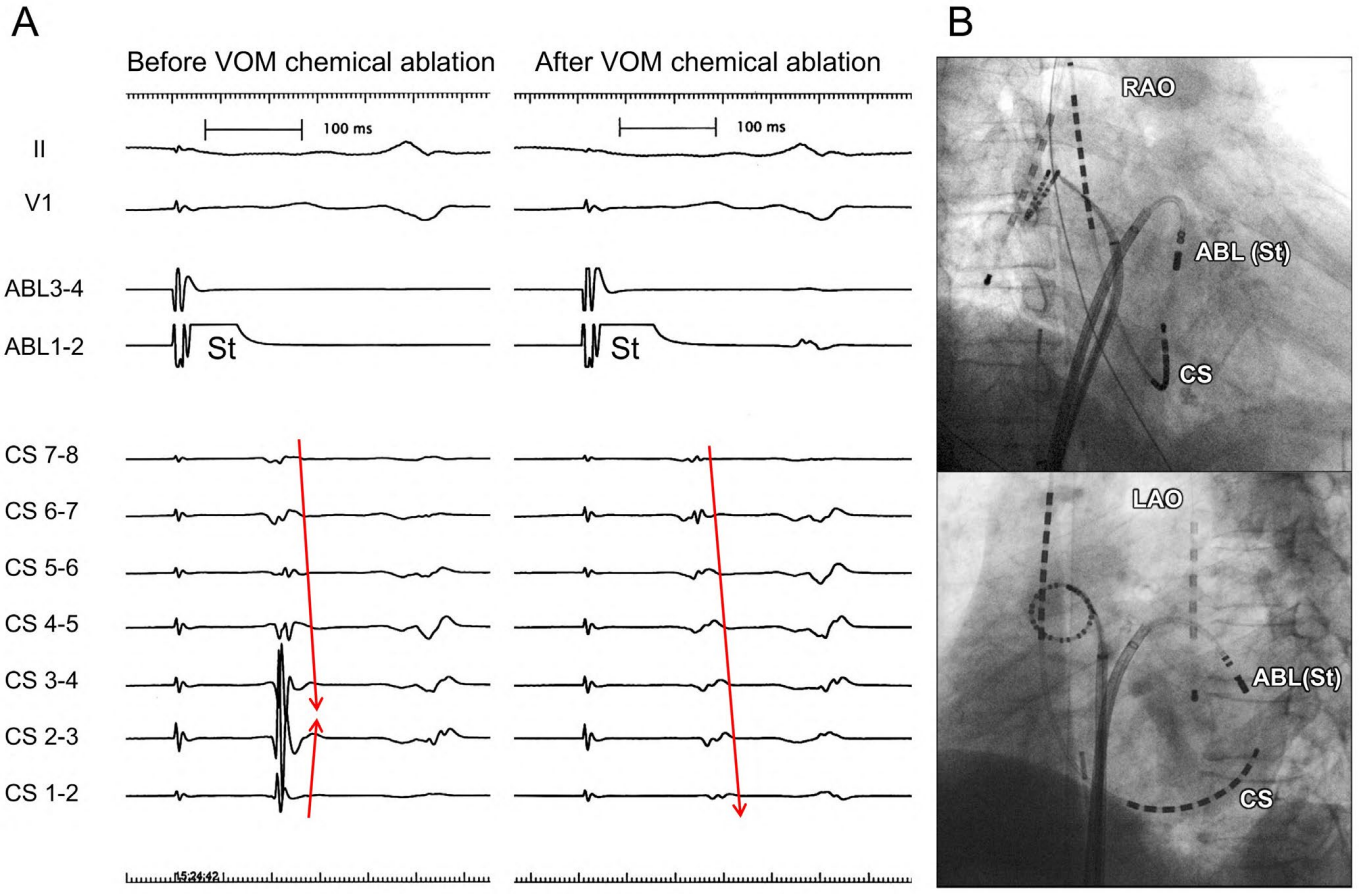
Mitral isthmus block creation. (A) Intracardiac electrogram before and after the chemical ablation of the VOM. During pacing from the ablation catheter positioned above the mitral isthmus line before chemical ablation of the VOM, the activation of the CS electrodes collided at CS 2–3 and 3–4. After VOM chemical ablation, the activation of the CS electrodes traveled in a proximal‐to‐distal direction, indicating the successful creation of a complete mitral isthmus block, as indicated by the red arrows. (B) Catheter position during mitral isthmus block. The ablation catheter was positioned superior to the mitral isthmus line, while the CS catheter was positioned inferior to it. ABL, ablation catheter; CS, coronary sinus; LAO, left anterior oblique; RAO, right anterior oblique; VOM, vein of Marshall.

**FIGURE 5:**
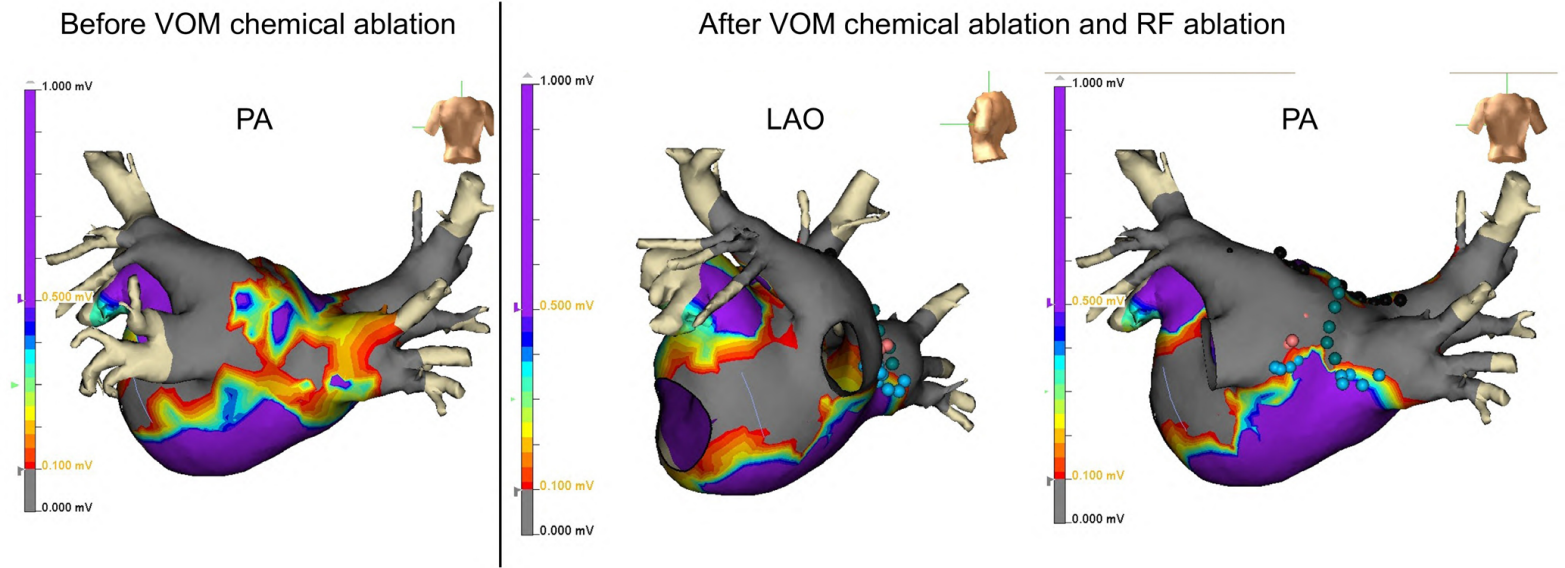
Voltage map before VOM chemical ablation and after both VOM chemical ablation and RF ablation. LAPW was not isolated prior to VOM chemical ablation. Complete isolation was achieved following additional centerline RF ablation, after VOM chemical ablation and RF ablation along the left atrial roof and floor. A complete scar lesion was observed in the mitral isthmus region. LAO, left anterior oblique view; LAPW, left atrial posterior wall; VOM, vein of Marshall; PA, posterior–anterior view; RF, radiofrequency.

VOM ethanol infusion achieves a high success rate in mitral isthmus block and enhances AF ablation outcomes. Haissaguerre et al. reported on the efficacy of adjunctive VOM chemical ablation, known as the “Marshall Plan.” In the VENUS trial, Valderrabano et al. showed that adjunctive VOM chemical ablation reduced AF recurrence in patients with persistent AF. Valderrábano et al. [4] examined the effects of VOM ethanol infusion on the intrinsic cardiac nerves involved in AF. They reported that VOM chemical ablation suppressed parasympathetic responses in the region, thereby reducing AF inducibility.

Keida et al. [5] reported a case in which VOM chemical ablation eliminated frequent APCs with a non‐PV trigger initiating AF. Due to an insufficient activation map, the precise origin of the non‐PV trigger could not be identified. They concluded that VOM chemical ablation might either eliminate non‐PV foci arising from or near the VOM or modulate arrhythmogenic autonomic innervation in the region. Ascione et al. [6] reported that pulsed field ablation failed to isolate LAPW, but isolation was achieved incidentally with VOM chemical ablation intended for mitral isthmus block, suggesting a VOM branch perfusing the LAPW region. In our case, AF originating from the LAPW was terminated by VOM chemical ablation, with no recurrence. However, voltage mapping during sinus rhythm revealed that the LAPW remained electrically connected, making the extent of the ablation's effect unclear. Given that AF terminated immediately following ethanol injection and that the subsequent arrhythmia manifested as AT rather than recurrent AF, ethanol exerting a direct effect on the AF trigger is more plausible than the termination being attributable to a vagal response. Moreover, VOM angiography showed small branches extending toward the LAPW, suggesting that VOM chemical ablation may have partially ablated the non‐PV trigger substrate in the LAPW. Valderrábano et al. reported that ethanol infusion into the VOM may modulate autonomic ganglia in the left atrium, particularly around the Marshall bundle, which are known to influence atrial electrophysiology and may contribute to the observed changes in LAPW activity and the suppression of AF [7]. Furthermore, the distal portion of the VOM, which courses epicardially along the left atrial ridge toward the posterior wall, may be particularly relevant in mediating these effects through both direct tissue injury and autonomic modulation. Yokoyama et al. reported that the distal portion of the VOM, which courses epicardially along the left atrial ridge toward the posterior wall, contains ganglionated plexi (GP), including the inferior left GP and the ligament of Marshall GP. These autonomic structures may play a role in the initiation and maintenance of AF [8]. In our case, mitral isthmus ablation failed during the first session despite additional RF intra‐CS ablation. However, VOM chemical ablation in the second session successfully achieved a bidirectional mitral isthmus block. This case illustrates that although VOM chemical ablation is an established strategy for achieving mitral isthmus block, it may also be effective in treating non‐PV‐triggered AF, thereby providing dual benefits and effectively “killing two birds with one stone.”

This report has few limitations. This is a single‐case report, and detailed voltage mapping during sinus rhythm was not performed to delineate the accurate extent of LAPW modification. The absence of intracardiac echocardiography or high‐density mapping to visualize conduction gaps, along with the lack of autonomic testing to support claims of GP modulation, further limits the mechanistic interpretation. The involvement of GP and the contribution of VOM branches to AF elimination remain speculative, limiting the ability to generalize these findings. This case was considered to originate from the LAPW, as the cycle length of the LAPW potentials was markedly shorter than in other regions at the onset of AF. Although the CFAE‐like signals observed during tachycardia are often associated with non‐PV triggers, it should be noted that CFAEs in the LAPW are not specific to AF triggers and may also occur in areas of slow conduction or structural remodeling, limiting their diagnostic value. Nevertheless, to our knowledge, this is the first report demonstrating that VOM chemical ablation can simultaneously achieve mitral isthmus block and eliminate LAPW‐origin non‐PV triggers, suggesting its potential value in complex non‐PV AF scenarios.

## Ethics Statement

This study was approved by the Japan Redcross Yokohama City Bay Hospital Ethics Committee.

## Consent

Written informed consent was obtained from the patient.

## Conflicts of Interest

The authors declare no conflicts of interest.

## Data Availability

The data that supports the findings of this study are available in this article.
